# Price Variation in Outpatient Percutaneous Coronary Intervention

**DOI:** 10.1161/CIRCULATIONAHA.126.081539

**Published:** 2026-10-27

**Authors:** Kriyana P. Reddy, Navneeth Murali, Nicholas K. Smith, Sameed Ahmed M. Khatana, Paula Chatterjee, Alexander C. Fanaroff, Peter W. Groeneveld, Jay Giri, Ashwin S. Nathan

**Affiliations:** 1 Penn Cardiovascular Quality, Outcomes, and Evaluative Research Center (K.P.R., N.M., N.K.S., S.A.M.K., A.C.F., P.W.G., J.G., A.S.N.), University of Pennsylvania 6572 , Philadelphia.; 2 Divisions of Cardiovascular Medicine (N.K.S., S.A.M.K., A.C.F., J.G., A.S.N.), University of Pennsylvania 6572 , Philadelphia.; 3 General Internal Medicine (P.C., P.W.G.), University of Pennsylvania 6572 , Philadelphia.; 4 Perelman School of Medicine, and Leonard Davis Institute of Health Economics (S.A.M.K., P.C., A.C.F., P.W.G., J.G., A.S.N.), University of Pennsylvania 6572 , Philadelphia.; 5 Corporal Michael J. Crescenz VA Medical Center, Philadelphia, PA (S.A.M.K., P.W.G., J.G., A.S.N.).

**Keywords:** costs and cost analysis, outpatients, percutaneous coronary intervention, price transparency

More than 600 000 percutaneous coronary interventions (PCIs) are performed annually in the United States.^1^ Increasing procedural safety, same-day discharge pathways, and expansion of Centers for Medicare & Medicaid Services reimbursement have accelerated the transition of elective PCI into outpatient settings, including hospital outpatient departments (HOPDs) and ambulatory surgical centers (ASCs).^2–4^ Although outpatient PCI may reduce resource utilization and overall spending, little is known about how negotiated prices vary across outpatient sites of care.^2^

The 2021 Hospital Price Transparency Rule requires hospitals to disclose payer-specific negotiated rates for all services and has enabled national analyses of pricing variation for cardiovascular services.^5^ Previous work leveraging these data has demonstrated substantial variation in commercial prices for common cardiovascular procedures, but outpatient PCI pricing remains poorly characterized.^5^ We used national price transparency data to quantify variation in prices for outpatient PCI across HOPDs versus ASCs and to evaluate associations between facility characteristics and negotiated commercial prices.

## Methods

This study was exempt from review by the institutional review board at the University of Pennsylvania. Data used for this study are covered by a Data Use Agreement and are not available for distribution by the authors. Price data were obtained from Turquoise Health, which standardizes payer-provided machine-readable files into structured data by harmonizing billing codes and prices across heterogeneous file formats.^6^ We identified commercial and Medicare prices associated with Current Procedural Terminology code 92928 between April 1, 2025, and July 31, 2025. Medicare prices were derived by Turquoise Health using Centers for Medicare & Medicaid Services Inpatient Prospective Payment System/Outpatient Prospective Payment System hospital prices and ASC fee schedules. Commercial prices may not include device or implant costs because some contracts use device carve-outs not identifiable in the available data. Therefore, direct comparisons between Medicare and commercial prices were not performed.

Facilities were classified as HOPDs or ASCs using National Uniform Claim Committee Taxonomy codes. Analyses were limited to facilities with ≥1 commercial and ≥1 Medicare price, excluding facilities unlikely to perform PCI using predefined keyword matching. Prices flagged as outliers by Turquoise Health as well as prices 1.5 interquartile ranges below the first quartile or above the third quartile within each payer class were excluded. Data from Dartmouth Atlas, US Census Bureau, and Health Care Cost Institute were used to define hospital referral region (HRR), Census region, metropolitan status, and market concentration.

Prices were summarized using medians (interquartile ranges) and ranges. Within-facility commercial price variation was quantified as the ratio of maximum to minimum commercial price. The primary aim was to compare price differences between HOPDs and ASCs. First, price distributions by facility type were compared using Kruskal-Wallis tests. Second, across-facility variation between HOPD and ASC prices was assessed using ratios of median HOPD-to-ASC prices within each HRR. Third, the association between facility type and median commercial prices was evaluated using a generalized linear mixed-effects model with a gamma distribution, log link, random intercepts for HRR, and adjustment for facility type, Census region, metropolitan status, and market concentration. The gamma-log specification was selected given positive and right-skewed prices and because variance was found to increase approximately with the square of the conditional mean (modified Park test variance of power, 1.68 [95% CI, 1.31–2.04]), consistent with a gamma-type variance function. Previous methodologic work recommends selecting models based on observed mean-variance relationship, and empirical comparisons have demonstrated favorable performance of gamma-log models for skewed cost data.^7,8^ Analyses were performed using R version 4.4.1 with 2-sided *P*<0.05 considered statistically significant.

## Results

Across 8486 outpatient facilities, we identified 44 871 prices for Current Procedural Terminology code 92928, including 36 285 commercial and 8586 Medicare prices. Commercial prices varied substantially, with a median (interquartile range) of $4149 ($980–$11 460). Medicare prices were comparatively uniform, with a median (interquartile range) of $10 787 ($7103–$11 382). Median Medicare prices were significantly higher in HOPDs than ASCs ($11 341 versus $6874; *P*<0.001). Within-facility commercial price variation was greater among HOPDs than ASCs, with median maximum-to-minimum commercial price ratios of 6.27 versus 2.21, respectively (*P*<0.001; Table). Across HRRs, median HOPD-to-ASC commercial price ratios exceeded 10 in some markets, whereas Medicare price ratios were generally <2 (Figure 1). In the adjusted generalized linear mixed-effects model with HRR-level clustering, ASCs had 26% lower median commercial prices than HOPDs (rate ratio, 0.74 [95% CI, 0.7–0.78]; *P*<0.001). Facilities in micropolitan areas also demonstrated lower commercial prices than metropolitan facilities (rate ratio, 0.39 [95% CI, 0.17–0.92]; *P*=0.032).

**Table.**
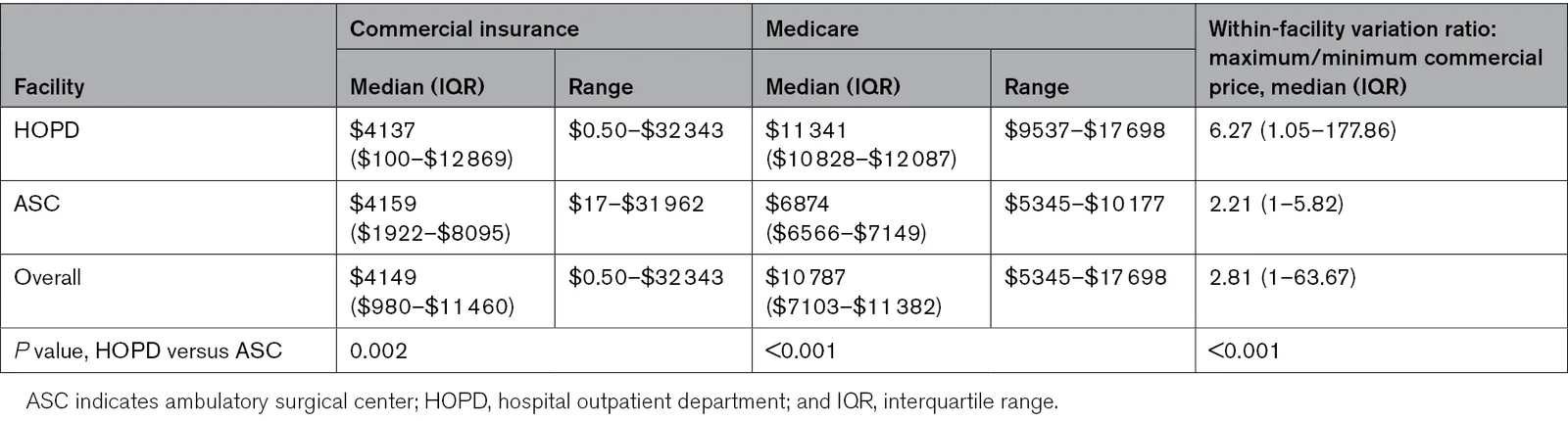
Variation in Commercial and Medicare Prices for Current Procedural Terminology Code 92928

**Figure.**
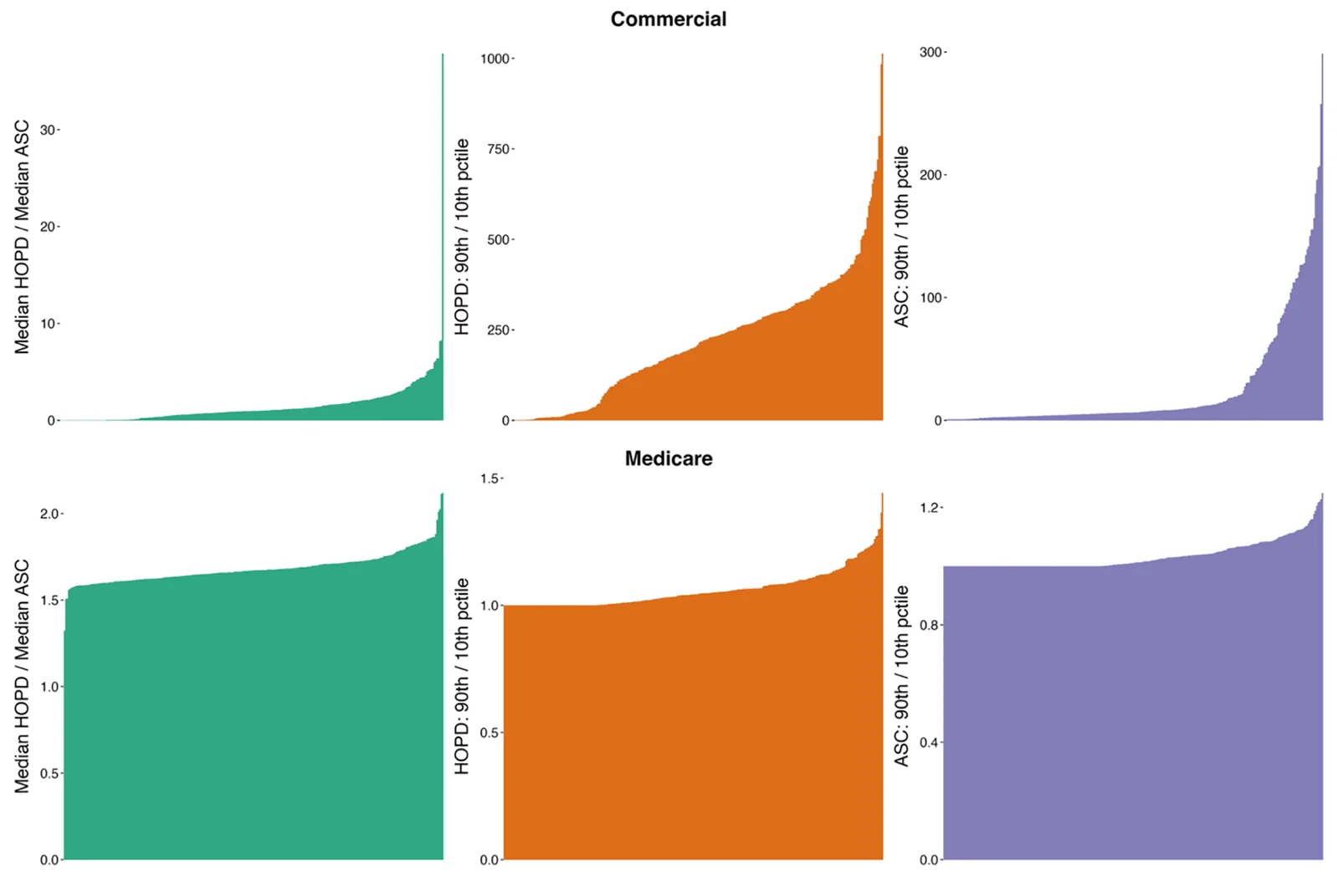
**Across-facility variation in commercial and Medicare prices for Current Procedural Terminology code 92928.** Each bar represents 1 hospital referral region.

## Discussion

In this national analysis of >8000 outpatient facilities reporting prices for PCI, commercial prices demonstrated substantial variation. Within-facility commercial price variation was greater among HOPDs than ASCs, with median maximum-to-minimum price ratios of 6.27 versus 2.21. After adjustment for geography, metropolitan status, and market concentration, ASCs had 26% lower negotiated commercial prices than HOPDs.

These findings highlight important differences between administratively determined Medicare reimbursement and commercially negotiated pricing. Medicare prices clustered within narrow ranges across facilities, whereas commercial prices varied widely both within and across outpatient settings. HOPDs demonstrated significantly greater within-facility variation than ASCs, which may reflect more complex hospital contracting across multiple payers and products. In contrast, ASCs may use more standardized contracting structures across a narrower portfolio of services and payers. Lower prices among ASCs may additionally reflect lower operational and infrastructure costs relative to HOPDs, which generally maintain greater staffing, regulatory, and hospital-based support requirements and may care for more clinically complex patients.^9^

Our findings may also have implications for ongoing discussions regarding site-neutral payment reform and expansion of outpatient cardiovascular care. Previous studies have demonstrated safety and feasibility of outpatient PCI in selected patients, and ASCs may represent a lower-cost setting for appropriately selected elective procedures. PCI was not included among the high-volume service categories proposed by MedPAC for site-neutral payments in 2023, yet the substantial ASC–HOPD commercial price differences observed in this study suggest potential opportunities to reduce unwarranted spending variation and generate savings as outpatient cardiovascular spending continues to rise.^10^ Simultaneously, payment reform should consider differences in patient complexity, procedural risk, and quality outcomes across outpatient settings. State-level regulatory frameworks, including certificate-of-need laws and restrictions on ASC PCI programs, may also limit the extent to which potential savings from site-neutral payment policies can be realized uniformly across markets.

This study has several limitations. Price transparency data may incompletely capture facilities performing PCI because of variable compliance with federal reporting requirements and heterogeneous reporting practices. Some extreme commercial prices may reflect differences in contract structure or reporting rather than true reimbursement differences. In addition, commercial contracts may differ in the degree to which device-related costs are bundled within reported prices, thereby limiting direct comparisons between commercial and Medicare prices. Analyses were limited to Current Procedural Terminology code 92928, which represents standard outpatient stent-based PCI and the largest share of posted PCI prices in the database, but findings may not generalize to other PCI procedures. Furthermore, posted prices do not capture professional fees or full episode-level reimbursement. Professional reimbursement represents a distinct payment structure that may independently influence physician incentives regarding site of care and warrants further investigation. We also could not account for differences in case mix, procedural acuity, or facility PCI volume, which may contribute to price differences between ASCs and HOPDs.

## Conclusion

Commercial prices for outpatient PCI vary substantially across HOPDs and ASCs, whereas Medicare prices are relatively uniform. HOPDs demonstrate greater commercial price variation and higher negotiated prices than ASCs. These findings may inform ongoing discussions regarding site-neutral payment reform and efforts to reduce unwarranted variation in outpatient cardiovascular spending.

## Article Information

### Disclosures

None.

## References

[1] Masoudi FA Ponirakis A de Lemos JA Jollis JG Kremers M Messenger JC Moore JWM Moussa I Oetgen WJ Varosy PD. Trends in U.S. cardiovascular care: 2016 report from 4 ACC national cardiovascular data registries. J Am Coll Cardiol. 2017;69:1427–1450. doi: 10.1016/j.jacc.2016.12.005 10.1016/j.jacc.2016.12.005 28025065

[2] Rymer JA O’Donnell CI Plomondon ME Hess PL Donahue M Hebert PL Shroff A Swaminathan RV Waldo SW Seto AH. Same-day discharge among patients undergoing elective PCI: insights from the VA CART Program. Am Heart J. 2019;218:75–83. doi: 10.1016/j.ahj.2019.09.003 10.1016/j.ahj.2019.09.003 31707331

[3] Box LC Blankenship JC Henry TD Messenger JC Cigarroa JE Moussa ID Snyder RW Duffy PL Carr JG Tukaye DN. SCAI position statement on the performance of percutaneous coronary intervention in ambulatory surgical centers. Catheter Cardiovasc Interv. 2020;96:862–870. doi: 10.1002/ccd.28991 10.1002/ccd.28991 32406995

[4] Centers for Medicare & Medicaid Services. Medicare program: changes to hospital outpatient prospective payment and ambulatory surgical center payment systems and quality reporting programs; revisions of organ procurement organizations conditions of coverage; prior authorization process and requirements for certain covered outpatient department services; potential changes to the laboratory date of service policy; changes to grandfathered children’s hospitals-within-hospitals; notice of closure of two teaching hospitals and opportunity to apply for available slots. Fed Regist. 2019;84:61142–61492.

[5] Reddy KP Mehta S Eberly LA Khatana SAM Chatterjee P Fanaroff AC Groeneveld PW Giri J Nathan AS. Inter- and intrahospital price variation for common cardiovascular admission diagnoses, diagnostic tests, and therapeutic procedures. J Am Heart Assoc. 2025;14:e038660. doi: 10.1161/JAHA.124.038660 10.1161/JAHA.124.038660 PMC12132732 40094183

[6] Dorstel M. Everything you need to know about the July 1, 2022, payer rates disclosure. Turquoise Health. Accessed May 17, 2026. https://turquoise.health/resources/blog/everything-you-need-to-know-about-the-july-1-2022-payer-rates-disclosure

[7] Manning WG Mullahy J. Estimating log models: to transform or not to transform? J Health Econ. 2001;20:461–494. doi: 10.1016/s0167-6296(01)00086-8 10.1016/s0167-6296(01)00086-8 11469231

[8] Dodd S Bassi A Bodger K Williamson P. A comparison of multivariable regression models to analyse cost data. J Eval Clin Pract. 2006;12:76–86. doi: 10.1111/j.1365-2753.2006.00610.x 10.1111/j.1365-2753.2006.00610.x 16422782

[9] Reddy KP Nathan AS Giri J. Economics of emerging transcatheter cardiovascular interventions. JACC Cardiovasc Interv. 2025;18:2169–2174. doi: 10.1016/j.jcin.2025.06.032 10.1016/j.jcin.2025.06.032 40772910

[10] Levinson Z Neuman T Hulver S. Five things to know about Medicare site-neutral payment reforms. KFF. Accessed March 1, 2026. https://www.kff.org/medicare/five-things-to-know-about-medicare-site-neutral-payment-reforms

